# Symptomatic Apical Periodontitis of the Mandibular First Molar with the Accessory Canal in the Furcation Area Mimicking Furcation Perforation

**DOI:** 10.1155/2022/6324447

**Published:** 2022-05-12

**Authors:** Alexei Bolyachin, Zurab Khabadze, Oleg Mordanov, Magomed Gasbanov, Takhir Teberdiev

**Affiliations:** ^1^Iceberg Dental Trauma Center, Moscow, Russia; ^2^RUDN University (People's Friendship University of Russia), Moscow, Russia

## Abstract

Apical periodontitis frequently presents as a chronic disease. To arrive at a true diagnosis, in addition to the clinical examination, it is mandatory to undertake radiographic examinations and evaluate the clinical presentation. Knowledge of the root canal morphology is a prerequisite for effective nonsurgical endodontic treatment. The internal morphological features of the pulp chamber are variable and complex. This case report describes the treatment and outcome of symptomatic apical periodontitis of a mandibular first molar with the accessory (chamber) canal. The applied treatment fully contributed to the periapical lesion regression as shown in the four-year recall periapical radiography.

## 1. Introduction

Apical periodontitis is mainly a root canal infection, characterized by inflammation and destruction of periapical and periradicular tissues resulting from the interaction between microbial factors and the host immune response [1, 2]. Destruction of the periodontal ligament is triggered by degradation of the extracellular matrix by metalloproteinases (MMPs) [3] involving periradicular inflammation and bone destruction mediated by proinflammatory cytokines [4].

Normal oral microorganisms and bacteria may give rise to opportunistic pathogens if access to dental pulp tissues appears. Openings in the physical dentin barriers (enamel and cementum) by means of caries, cracks, or traumatic injuries create pathways for bacteria into the pulp chamber and root canal system consequently [5].

Communication between the pulp and the periodontal ligament may occur via the apical foramen and lateral and accessory canals in the apical and coronal parts of the root. Despite careful selection of cases and the use of correct procedures, endodontic treatment occasionally fails, and it can be due to the presence of communications between the pulp tissues and periodontal tissues [6].

Accessory canals result due to a localized failure in the formation of Hertwig's sheath during the embryonic stages of tooth formation. This defect is probably due to the persistence of abnormally placed blood vessels reaching the pulp, which is more common in the furcation region [6].

**Figure 1:**
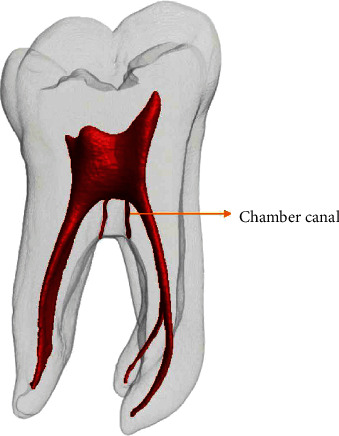
Schematic representation showing chamber canals.

These canals are also known as interradicular canals [7]. In 2018, Ahmed et al. [8] suggested a new accessory canal classification. The proposed classification provides an accurate description of the position and configuration of the accessory and chamber canals. By chamber canals, the authors mean small canals leaving the pulp chamber that (usually) communicates with the external surface of the root (including the furcation) (Figure 1).

The aim of this case report is to present the treatment and healing of apical chronic periodontitis with isolated furcation bone and apical bone destruction.

## 2. Case Report

A 27-year-old female patient with noncontributory general medical history presented and was referred to the dental practice in Moscow, Russia. The patient presented to the clinic with the complaint that included nonacute pain forcing on the tooth load during mastication from tooth # 46.

Pulp testing with carbon dioxide did not show any expressive reaction. Percussion was positive. Periodontal probing depth was 3 mm in average.

**Figure 2:**
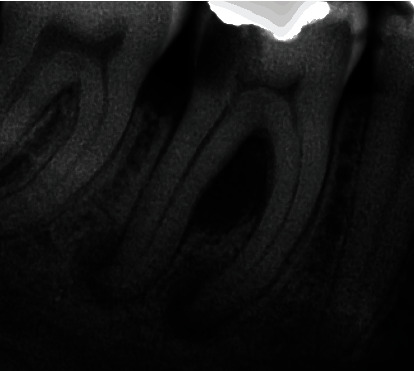
Apical radiograph of tooth # 46. Isolated furcation bone and apical bone destruction in the mesial and distal roots.

Periapical radiograph revealed a deep carious lesion in the distal surface of the tooth, communicating with the pulp chamber; there is presence of a radiolucency in the furcation region, not connected with the apical bone lesions present in both roots. No endodontic treatment had been previously performed (Figure 2).

The pulp chamber floor examination with the operative microscope (Carl Zeiss Meditec AG, Jena, Germany) with different zooming revealed no furcation, perforation, or fracture.

The following treatment strategy was applied. Coronal root canal portion was instrumented with Protaper SX (Dentsply Maillefer, Ballaigues, Switzerland) following a glide path development with C-Pilot (VDW, Munich, Germany) 6-15 ISO sizes. The shaping was provided with FlexMaster (Vereinigte, Dentalwerke, Munich, Germany) 15-35 ISO sizes, 4% tapered.

Irrigation protocol included the following: 3% NaOCl, 19% EDTA with the EndoActivator System (Dentsply Tulsa Dental Specialties, Tulsa, OK, USA). Pulp chamber irrigant solution was intensively activated with the ultrasound E4 tip, 3 cycles. Every cycle time was 30 seconds with 30% of ultrasound power in the general mode.

The first appointment was finished placing a calcium hydroxide-based medication on the working length (UltraCal XS paste; Ultradent Products, Inc., South Jordan, UT, USA). The floor of the pulp was covered with polytetrafluoroethylene (PTFE) and retained with the thin composite resin layer (Filtek Flow, 3M ESPE, Saint Paul, MN, USA) for the better sealing and retention. The outer part of the cavity was restored with intermediate restorative material (IRM) according to the double seal technique [9].

**Figure 3:**
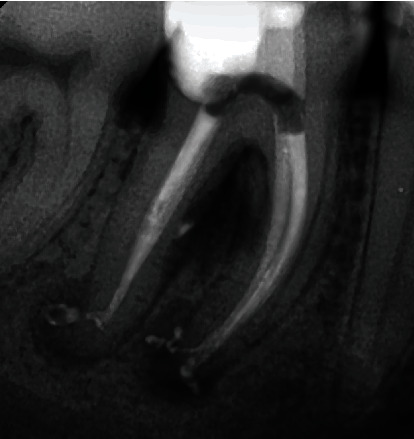
Apical radiograph of tooth # 46 after obturation.

After two weeks, the medication was removed with the instrumentation with FlexMaster and irrigation with 3% NaOCl. As the patient was asymptomatic, the canals were dried and filled. The obturation was performed using continuous wave technique with gutta-percha and AH Plus (Dentsply International Inc., York, PA, USA) (Figure 3).

**Figure 4:**
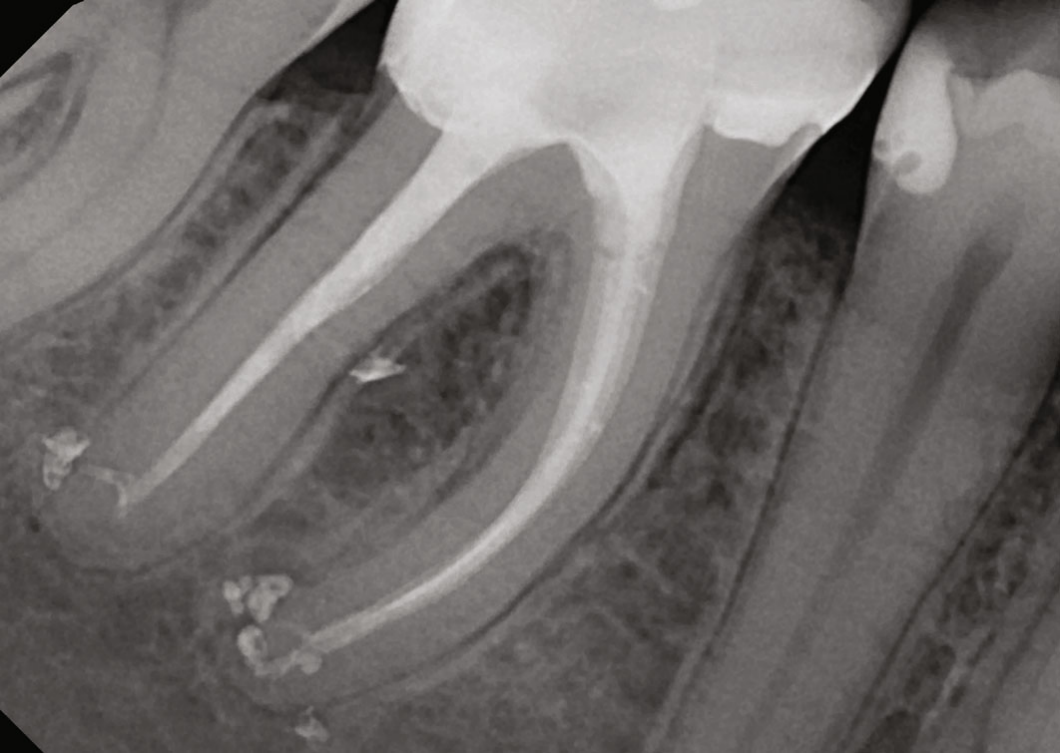
Two-year follow-up visit. Full healing was observed in the furcation and the periapical areas of tooth # 46.

**Figure 5:**
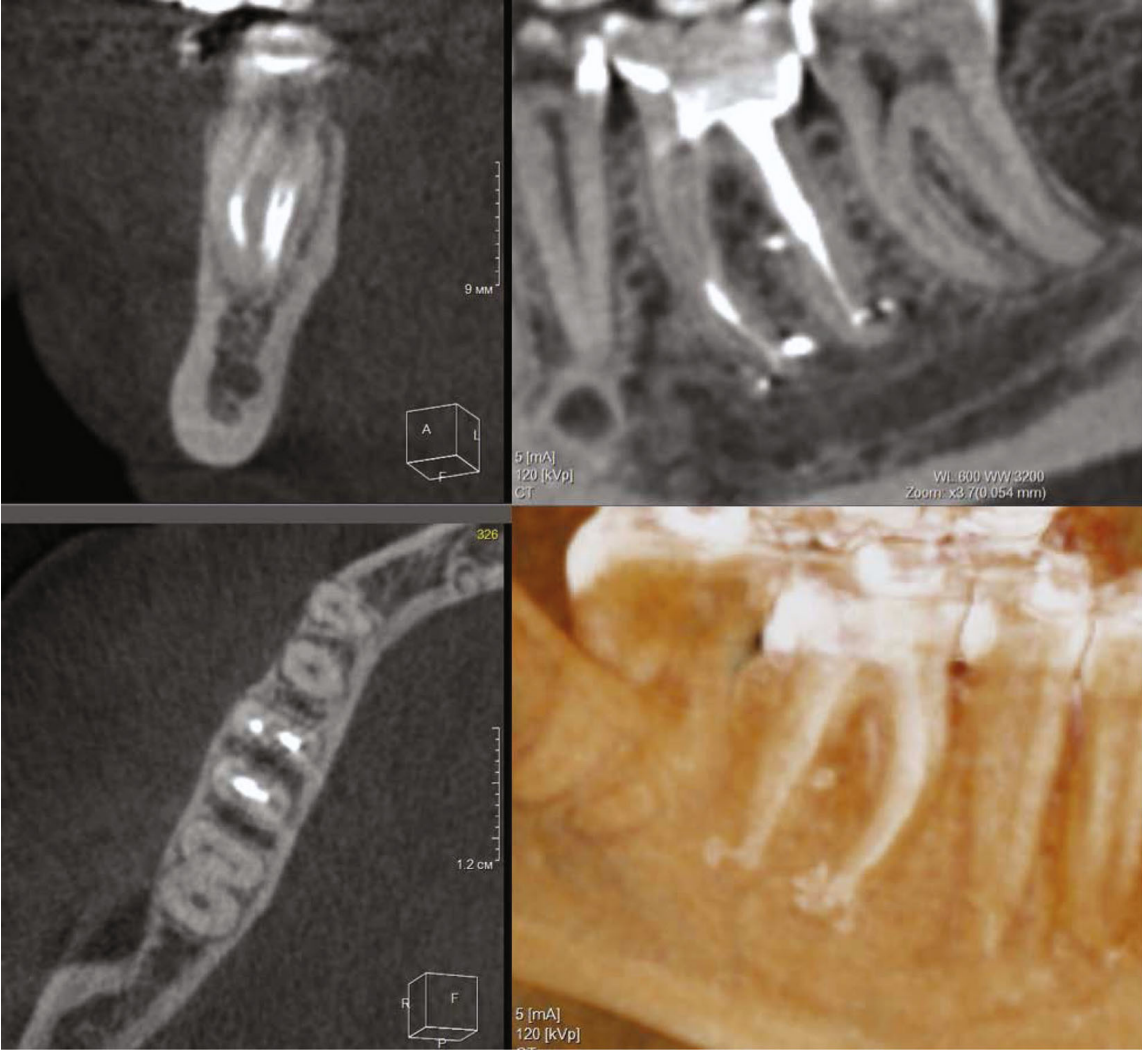
CBCT scan of tooth # 46 in 48 months after obturation. Full bone recovery is noticed.

After obturation, the patient was referred to the restorative treatment. The tooth was restored with a lithium disilicate full crown. The patient was recalled after 6, 12, 24, and 48 months (Figure 4). Total periapical and furcation healing was observed after 12 months and confirmed by the Cone Beam Computed Tomography (CBCT) scan 48 months after obturation (Figure 5).

## 3. Discussion

Different researches aimed at observing interradicular canals in lower molars have reported similar or different results, methodologies, terminology definition, and sample numbers [10].

Wolf et al. [10] observed 9 (7.7%) interradicular canals communicating the pulp chamber floor with the bifurcation area using dye in extracted teeth. The authors investigated the pulp chamber floor and the bifurcation area surface of 117 mandibular first molars. Access cavities were prepared; the pulp chambers were flooded with methylene blue and then centrifuged. An average of 4.2 (0.145 ± 0.03 mm thickness) slices per tooth were obtained by means of a diamond band saw. The presence of interradicular canals and diverticula was investigated using a light microscope (125x). Perlich et al. [11] found 3 canals (4.8%) using scanning electron microscopy and 22 canals (64.5%) using light microscopy in the pulp chamber floor of 62 human maxillary and mandibular molars. Chouchi et al. [12] studied accessory canals in 57 extracted permanent human teeth with micro-CT and revealed 7% (*n* = 4) and 21% (*n* = 12) furcation canals in the first and second mandibular molars, respectively.

Furcation perforation is an injury that leads to a communication between the root canal and the periodontal tissues or the oral cavity. It can be caused by resorption or dental caries or can have an iatrogenic cause including the misaligned use of rotary burs during access, preparation, and search for the root canals [13]. Furcation involvement refers to the condition when periodontal disease has caused bone resorption at the bifurcation or trifurcation of a multirooted tooth [14, 15]. Clinical examination with magnification, vital tests, and no previous endodontic treatment can help to exclude furcation impairment by periodontal disease.

Successful endodontic treatment depends on the thorough debridement of the entire root canal system. Difficult canal anatomy could be one of the causes of improper cleansing of the canal system, with the permanence of microorganisms [16, 17].

Irrigation of the root canals plays an important role in the disinfection process. From a microbiological standpoint, an irrigant should be able to demonstrate antimicrobial and antibiofilm activities and inactive endotoxins [18]. Activation of the irrigant helps to improve root canal cleanliness [19, 20]. Al-Jadaa et al. [21] demonstrated that ultrasonic irrigation causes a rise in the irrigant temperature in the accessory canals to 53.5 ± 2.7°C after the fifth minute, which could improve the irrigation effectiveness. No significant influence of accessory canal position or angulation was found.

Temporary restorative materials are often used during endodontic treatment to seal the root chamber between sessions or until a permanent restoration is placed. An ideal temporary restorative material should exhibit no leakage, good abrasion and compression resistance, lack of porosity, and lack of dimensional changes, and it must also be easily manipulated or removed while being effective in a moist environment [22]. PTFE is also an inexpensive material and previously used in dentistry in different fields. PTFE tape did not provide an avenue for bacterial contamination as temporary spacer materials [23]; however, PTFE retained by friction and may not be applicable in some other conditions, so it needs to be retained with stable material, such as the lite-cured composite resins [24]. E. faecalis grows on and penetrates through PTFE significantly more than it does with IRM (the most commonly used temporary restorations among specialists [24]), revealing its comparably reduced overall antimicrobial sealing ability when placed as the base part of temporary restorations [25]. Furthermore, research has shown that IRM has a poor marginal seal, performs poorly under stress, and exhibits bacterial penetration through the bulk of the material [26]; that is why composite resin creates extra the protection layer from leakage. The use of the composite resin as a full external layer increases the cost of the endodontic treatment.

The “double seal” technique [9] involves placing composite resin as the deeper layer material inside the pulp chamber and access cavity. The IRM is then used as the outer layer which is exposed to loading and the oral cavity. This double layer functions in several ways: the outer layer of IRM is an antibacterial agent; the inner layer of composite resin prevents liquids from the oral cavity to reach the root canal system if it has been able to penetrate through the IRM margins. The white colour of the IRM is readily visible when the clinician needs to remove it. The IRM is also a cheap material that is easily and quickly mixed and placed in the tooth. It sets quickly, and therefore, there is no “waiting time” after placement before the rubber dam can be removed.

## 4. Conclusion

Activation of the irrigant associated with the instrumentation and the use of the intracanal medication could explain the repair of the furcation and periapical lesions after endodontic treatment.
